# GluK2-Mediated Excitability within the Superficial Layers of the Entorhinal Cortex

**DOI:** 10.1371/journal.pone.0005576

**Published:** 2009-05-18

**Authors:** Prateep S. Beed, Benedikt Salmen, Dietmar Schmitz

**Affiliations:** NeuroScience Research Center, Charité, Universitätsmedizin Berlin, Berlin, Germany; Instituto Cajal - CSIC, Spain

## Abstract

Recent analysis of genetically modified mice deficient in different kainate receptor (KAR) subunits have strongly pointed to a role of the GluK2 subunit, mediating the vulnerability of the brain towards seizures. Research concerning this issue has focused mainly on the hippocampus. However, several studies point to a potential role of other parts of the hippocampal formation, in particular the entorhinal cortex, in the development of epileptic seizures. There is extensive cell death after such seizures in layer III of the medial entorhinal cortex (LIII mEC), making this region of special interest for investigation into related pathological conditions. We therefore characterized KAR mediated currents in LIII mEC pyramidal neurons by several different approaches. Using patch-clamp technique, in combination with glutamate uncaging in horizontal brain slices, we show that LIII mEC neurons exhibit KAR currents. Use of genetically modified mice reveal that these currents are mediated by GluK2 containing KARs. The IV curve indicates the predominant presence of a Ca^2+^ impermeable and edited form of the KAR. Finally, we show that GluK2 containing kainate receptors are essential for kainate-induced gamma oscillations within the entorhinal cortex.

## Introduction

Kainate receptors (KARs) have a wide functional spectrum, ranging from the presynaptic regulation of transmitter release to the postsynaptic generation of excitatory inward currents [1], [2], [3]. Furthermore, there is evidence indicating that they are also involved in brain rhythmogenesis [4], [5], [6], [7], [8].

In contrast to alpha-amino-3-hydroxy-5-methyl-4-isoxazolepropionic acid receptors (AMPARs), which have been studied extensively, the roles and physiological importance of KARs are less well understood, although they were originally cloned and described over a decade ago [9], [10], [11], [12] and for reviews see [2], [3], [13]. One reason for this lack in our understanding of KAR function is the limited availability of pharmacological agents that enable KARs and AMPARs to be functionally distinguished. The generation of different KAR specific knock-out (KO) mice partially helped to overcome this drawback [14], [15] and their characterization yielded insights into KAR physiology. The recent development of the AMPAR selective antagonists GYKI 52466 and GYKI 53655 has also considerably advanced research in the KAR field.

One particular interesting aspect of KAR mediated action is the ability of the KAR agonist kainate, which exhibits binding preference for KARs, to evoke epileptic seizures following *in vivo* administration in mice [16]. The interpretation that KAR activation, rather than unspecific side effects due to activation of other glutamate receptors, is responsible for this phenomenon is supported by the fact that GluK2 KO mice have a much higher threshold for the induction of epileptic seizures [14] as compared to wild-type mice.

Epileptic seizures can also be evoked by electrical kindling of the entorhinal cortex or the perforant path (which leads to antidromic excitation of the entorhinal cortex, EC). For this reason, the EC is a prime candidate region for the development of temporal lobe epilepsy (TLE). The extensive interlaminar and intralaminar connectivity of the EC provide an ideal anatomical network for the generation of seizures [17]. Additionally, in the later stages of the development of epilepsy, the EC is one of the first brain regions to suffer from severe cell death. This holds especially true for LIII mEC, making this region of special interest for investigation into the related pathological conditions. Despite this, there has been relatively little research into the basic features of KAR mediated transmission in this region.

In this study we demonstrate the occurrence of KAR mediated currents in LIII mEC pyramidal neurons. These currents are conducted by GluK2 containing, Ca^2+^ impermeable receptors.

## Methods

### Slice preparation

Animal husbandry and experimental intervention were performed according to the german animal welfare act and the European Council Directive 86/609/EEC regarding the protection of animals used for experimental and other scientific purposes. All animal maintenance were performed in accordance with the guidelines of local authorities, Berlin [T 0100/03]). Wistar rats and C57/BL6 mice (2–3 weeks) were used for this study. The GluK1 and GluK2 mice used in this study were raised on a C57/BL6 background and littermate wildtype mice were used as control in such experiments. The animals were anaesthetized with isoflurane, decapitated and brains were rapidly removed and placed in ice-cold (4°C) oxygenated artificial cerebrospinal fluid (ACSF) containing (in mM): NaCl (87), NaHCO_3_ (26), Sucrose (75), Glucose (25), KCl (2.4), NaH_2_PO_4_ (1.25), MgCl_2_ (7), and CaCl_2_ (0.5), pH 7.4. Horizontal, combined entorhinal-hippocampal brain slices (300 µm; 400 µm for oscillation experiments) were cut by Leica VT 1200 Vibratome (Leica Microsystems, Wetzlar, Germany). Slices were then incubated at 34–35°C for 30 minutes and thereafter transferred to ACSF containing (in mM): NaCl (119), NaHCO_3_ (26), Glucose (10), KCl (2.5), NaH_2_PO_4_ (1.25), MgCl_2_ (1.3), and CaCl_2_ (2.5), at room temperature or to an interface-type recording chamber for oscillation experiments.. All ACSF solutions were equilibrated with carbogen (95% O_2_ and 5% CO_2_).

### Electrophysiological recordings

Prior to recording, slices were transferred to a submerged recording chamber (Luigs and Neumann, Ratingen, Germany) and perfused with oxygenated ACSF at room temperature, with a perfusion rate of of 2.5–3.0 ml/min. Recording electrodes of 2–3 MΩ resistance were pulled from borosilicate glass capillaries (Harvard Apparatus, Kent, UK; 1.5 mm OD) using a micropipette electrode puller (DMG Universal Puller). Biocytin (0.2%) was included (for a subset of recorded cells) in the patch pipette to assess the morphology and correct location of the recorded neurons following the experiments. The internal solution for all recordings included (in mM): K-gluconate (135), Hepes (10.0), EGTA (0.5), KCl (20), MgATP (2.0) and Phosphocreatine (5.0), with the exception of IV characterization, which included Cs-gluconate (140), Hepes (10.0), EGTA (1.0), CaCl_2_ (0.5), and Glucose (10.0).The osmolarities for the internal solutions were 300–305 mOsm, and the pH was adjusted to 7.2–7.3 with KOH or CsOH. Whole-cell voltage and current-clamp recording of LIII mEC pyramidal neurons were performed with an Axopatch 700A Amplifier (Axon Instruments, Union City, CA, USA). Data were acquired using a BNC-2090 adapter chassis, digitized (PCI 6035E A/D Board, National Instruments, Austin, Texas) at 5–10 kHz and recorded in IGOR Pro (WaveMetrics Inc., OR, USA).

Layer III mEC pyramidal neurons were initially recorded in the current-clamp mode to identify them according to their characteristic electroresponsive properties [18], [19]. Identified neurons were then held at −60 mV in voltage-clamp mode, and only recordings from neurons whose series resistances <30 MΩ were used for data analysis.

The whole-cell holding current experiments were done in the absence of any receptor-blockers unless mentioned otherwise on the figures.

Excitatory postsynaptic currents (EPSCs) were evoked by stimulating the layer I of the mEC (LI mEC). Synaptic KARs: Evoked EPSCs were recorded until the amplitudes of the responses were stable for a minimum of ten minutes before control and experimental drug application responses were obtained for analysis. EPSC_KA_ were pharmacologically isolated by adding 50 µM D(−)-2-amino-5-phosphonopentanoic acid (APV), 2 µM Gabazine (SR 95531), 20 µM SCH 50911, (Tocris, Ellisville, MO, USA), and 20 µM 1-(4-aminophenyl)-4-methyl-7,8-methylenedioxy-5H-2,3-benzodiazepine hydrochloride (GYKI 53655) to inhibit NMDA, GABA-A, GABA-B, and AMPA receptors respectively. GYKI resistant EPSCs were blocked by adding 25 µM 2,3-dioxo-6-nitro-1,2,3,4-tetrahydrobenzo(f)quinoxaline-7-sulfonamide (NBQX). A minimum of ten responses were recorded under each condition and averaged for analysis. Evoked EPSC amplitudes were calculated as the difference between the averaged peak response and the average of the baseline region (20 ms preceding the stimulus).

Glutamate uncaging: 20 ml of 200 µM MNI-caged-L-glutamate (Tocris, Bristol, UK) were reperfused at 2.5–3.0 ml/min. Uncaging was done using a UV pulsed laser (Rapp Optoelektronik, Wedel, Germany) attached with a 200 µm optical fiber coupled into the epifluorescence port of the microscope with an OSI-BX adapter (Rapp Optoelektronik, Wedel, Germany) and focused on the specimen by the objective lens. This yielded a illuminated circle (20–50 µm) covering the whole somatodendritic region of layer III cell bodies. The duration of the laser flash was 5 ms. The laser power under the objective corresponding to the stimulus intensity levels used was monitored using a photo diode array based photodetector (PDA-K-60, Rapp Optoelectronics, Wedel, Germany) and did not change over time. Glutamate was uncaged over the cell soma in the presence of all other channel blockers as mentioned above. In combined experiments where both the somatic and synaptic currents were recorded, first LI mEC was stimulated with a stimulation electrode thus evoking a synaptic response and after 200–300 ms, a laser pulse was flashed to uncage MNI-glutamate evoking somatic current.

For studying gamma oscillations, slices were stored and recorded from in the interface-type recording chamber. Extracellular recording electrode was placed in the superficial layers of mEC and baseline activity was recorded. Gamma oscillations were induced by bath applying 300 nM Kainic acid for upto 40 minutes.

### Morphology

A subset of electrophysiologically characterized LIII mEC pyramidal neurons were loaded with 0.2% biocytin and reconstructed for visualization. Slices were fixed overnight in 4% paraformaldehyde dissolved in 0.1 M sodium phosphate buffer (PB, pH 7.4) and incubated for 24 hours in PB supplemented with Avidin-coupled Alexa 488 (Invitrogen, Karlsruhe, Germany). After washing with PB the slices were dehydrated with ethanol, mounted on slides, and covered with DePeX (Serva, Heidelberg, Germany). Confocal laser scanning images were taken using a Leica TCS system (Bensheim, Germany).

### Statistical treatments

All values in all graphs are presented as mean+/−SEM. Analyses were performed using IGOR Pro (WaveMetrics Inc., OR, USA), SigmaPlot (SYSTAT, Hounslow, UK) and MATLAB v7.0 (The Mathworks Inc., MA, USA). Statistical comparisons between groups were performed with Student's t-test. Results were considered significant at *p*<0.05 or *p*<0.01 (*).

### Drugs

Kainic acid, (Kainate, KA); 1-(4-aminophenyl)-4-methyl-7,8-methylenedioxy-5H-2,3-benzodiazepine hydrochloride (GYKI 53655, henceforth in the manuscript GYKI would refer to GYKI 53655 unless mentioned otherwise); D(−)-2-amino-5-phosphonopentanoic acid (APV); 6-Imino-3-(4-methoxyphenyl)-1(6H)-pyridazinebutanoic acid hydrobromide (Gabazine); (2S)-(+)-5,5-Dimethyl-2-morpholineacetic acid (SCH 50911); 2,3-dioxo-6-nitro-1,2,3,4-tetrahydrobenzo(f)quinoxaline-7-sulfonamide (NBQX) and 6-Chloro-3,4-dihydro-3-(5-norbornen-2-yl)-2H-1,2,4-benzothiazidiazine-7-sulfonamide-1,1-dioxide (Cyclothiazide, CTZ) were all purchased from Tocris Bioscience (Ellisville, MO, USA).

## Results

**Figure 1:**
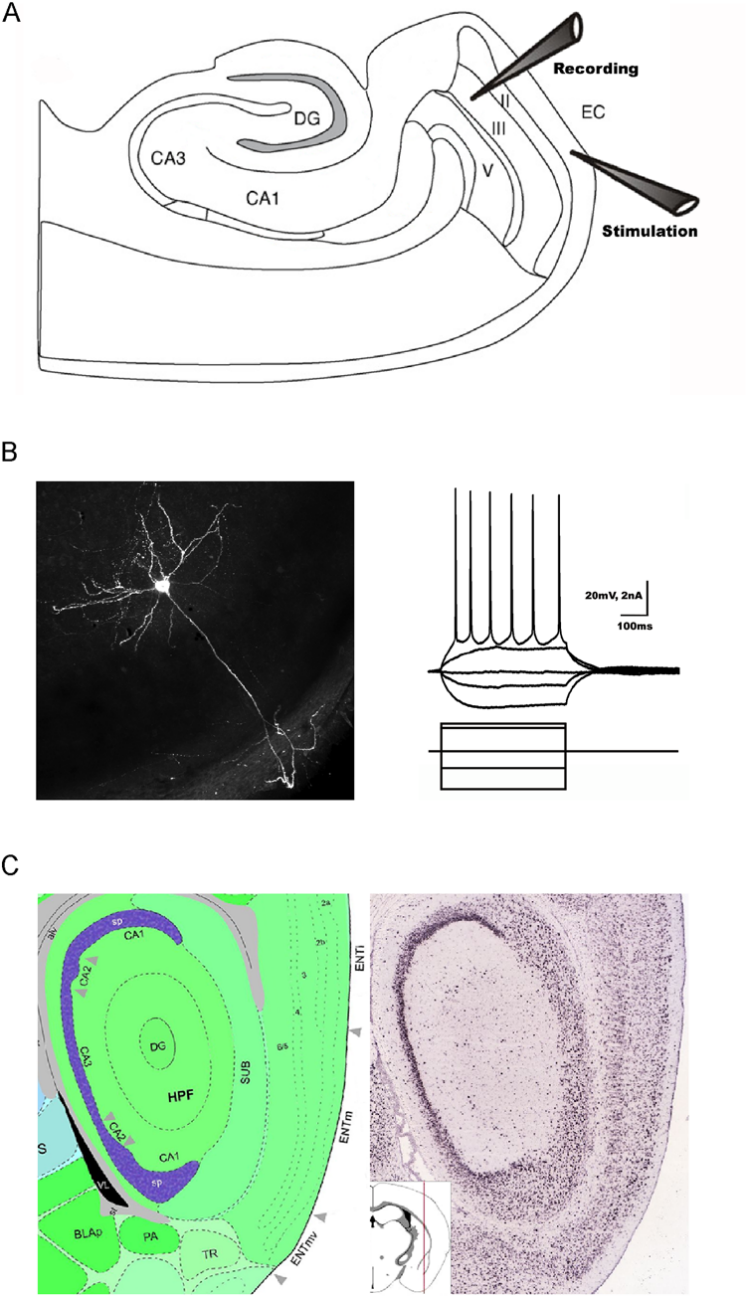
Layer III medial entorhinal cortex (LIII mEC) pyramidal neurons. (A) Schematic representation of the entorhinal-hippocampal combined slice used in this study with the recording electrode (Recording) in LIII mEC while stimulating (Stimulation in LI mEC) the input from the lateral entorhinal cortex. (B) Electrophysiological and morphological properties of a typical LIII mEC pyramidal neuron. (C) In situ hybridization of GluK2 subunit of kainate receptor in the mEC. Data adapted from the Allen Atlas, Allen Institute of Brain Science.

The entorhinal cortex is a six-layered cortical structure (Layers (L) I–V/VI; figure 1A) and the LIII mEC pyramidal neurons are easily distinguishable from all other entorhinal neurons based on anatomical location, morphology and characteristic electrophysiological properties ([18], [19], figure 1B). For this study we performed whole cell recordings from these neurons (figure 1A – experimental design). Furthermore, there is a mosaic like distribution of kainate receptors in the mEC both in terms of the subunit composition and layer-wise localization (figure 1C - GluK2 subunit; Adapted from the Allen Atlas, Allen Institute of Brain Science.), thereby offering an interesting prospect to study KAR mediated currents in the LIII mEC pyramidal neurons.

### Kainate concentration dependent changes in whole-cell holding current of LIII mEC pyramidal neurons

KA activates non-NMDARs (AMPARs and KARs) with different affinities. Low concentrations of KA (300 nM) activate only KARs while at higher concentrations (1 µM) it acts as an agonist for both AMPARs and KARs [14].

**Figure 2:**
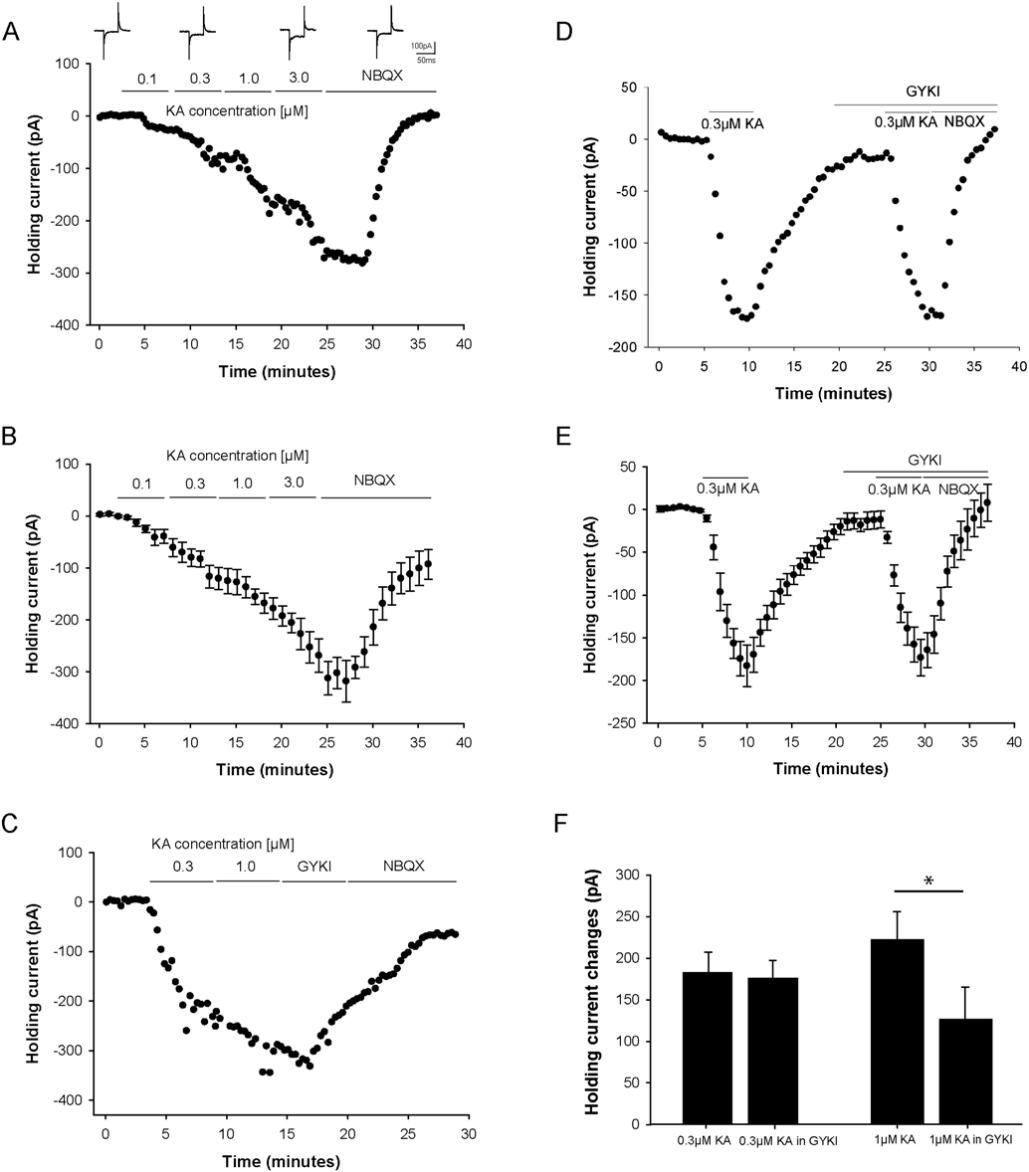
Kainate (KA) induced changes in whole-cell holding current and activation threshold of kainate receptors (KARs) on LIII mEC pyramidal neurons. (A, B) Time course data for KA (100 nM, 300 nM, 1 µM and 3 µM) induced concentration dependent changes in the whole-cell holding current which is antagonized by NBQX (25 µM). (A) Single experiment with voltage-clamp transients corresponding to baseline, 300 nM, 3 µM and NBQX. (B) Group data (n = 6). (C) Time course data from a single experiment for two different KA concentrations (300 nM and 1 µM) with subsequent application of GYKI (20 µM) and NBQX (25 µM). (D, E) Time course data for determining the activation threshold of kainate receptors on LIII mEC pyramidal neurons. The change in the whole-cell holding current by bath application of 300 nM of KA was reversible and following treatment with GYKI (20 µM), 300 nM KA was applied for a second time. Holding current decreased to the same amplitude indicating that at a concentration of 300 nM KA, no AMPA receptors are activated. (D) Single experiment. (E) Group data (n = 4). (F) While there is no effect of GYKI (20 µM) on holding current at 300 nM KA (*p* = 0.344; n = 4) there is a significant effect at 1 µM KA (*p*<0.05; n = 4).

Concentration dependent successive activation of non-NMDARs leads to conductance changes of the cell, reflected in corresponding changes in its holding current. After attaining whole-cell configuration (at −60 mV) a baseline of holding current was obtained, following which increasing concentrations (100 nM, 300 nM, 1 µM and 3 µM) of KA were bath applied for five minutes each. Further increase of KA leads to severely depolarized cells and unstable recordings and was therefore omitted. The changes in holding current (figure 2A - single cell with voltage-clamp transients corresponding to baseline, 300 nM, 3 µM and NBQX; 2B - group data, n = 6) at the end of the five minute KA bath application were 53.54±13.95 pA at 100 nM, 131.79±18.98 pA at 300 nM, 201.64±20.55 pA at 1 µM and 303.58±33.25 pA at 3 µM. The KA-induced holding current change was reduced after washing in 25 µM NBQX, indicating that non-NMDARs were involved.

### KAR mediated currents in LIII mEC pyramidal neurons

To study KAR mediated currents in LIII mEC pyramidal neurons it is necessary to isolate this current from the combined current mediated by AMPARs and KARs. Since KA activates both AMPARs and KARs at different concentrations, it was necessary for us to determine the particular KA concentration at which only KARs are activated. Inititially, two different KA concentrations (300 nM and 1 µM) were applied subsequently followed by GYKI (20 µM) while monitoring holding current changes (figure 2C). Since GYKI showed an effect on the holding current following the 1 µM KA wash-in, it suggested that at this concentration AMPARs are activated along with KARs. This is summarized below and in figure 2F. Therefore, to isolate a pure KAR-mediated change in holding current, only the lower concentration of 300 nM KA was bath applied in separate experiments. The holding current changed by 181.58±23.83 pA, following washout of KA, 20 µM GYKI was applied for 5 minutes and then 300 nM KA was reapplied (figure 2D–E, n = 4). On applying 300 nM KA for the second time, the holding current changed by 174±20.7 pA. Since the changes in holding current in the absence and presence of AMPA receptor blocker, GYKI was not significiantly different, these changes are therefore mediated predominantly by KARs (figure 2F, n = 4). At 1 µM KA, substantial amount of AMPARs were activated as there was a significant difference in holding current before (229.3±34.5 pA) and after (130.9±38.8 pA) washing in GYKI (figure 2F, n = 4). Since at 1 µM KA, AMPA receptors were also activated along with KARs, 300 nM KA was chosen as the working concentration which activated only KARs and no AMPA receptors for LIII mEC pyramidal neurons.

KAR-mediated currents were also analysed in LII mEC stellate neurons. We found smaller holding current changes following application of 300 nM KA, in the presence of GYKI, in stellate cells (108.8±15.4, n = 7) in comparison to pyramidal neurons (174±.20.7, n = 4) in layer III (figure S1B). However, this could be due to differences in membrane capacitance. Indeed, stellate cells have a smaller capacitance in comparison to layer III pyramidal neurons [20]. In a small number of neurons we analysed the current densities between the two neuronal populations, but could not detect any siginificant difference (LII stellate: 0.63±0.17 pA/pF; LIII pyramidal neurons: 0.75±0.08 pA/pF; p = 0.57; n = 3 for each cell type).

### GluK2 is the major subunit mediating the KAR current in LIII mEC pyramidal neurons

**Figure 3:**
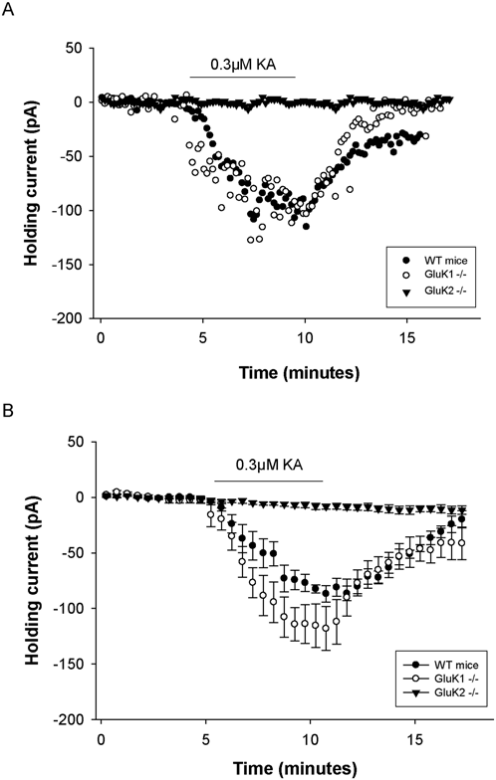
Genetic deletion studies to determine the role of GluK1 and GluK2 subunits in the KAR mediated current on the LIII mEC pyramidal neurons. (A, B) By bath applying 300 nM KA, the holding current in the GluK1 KO did not change significantly when compared to the wild-type mice (*p* = 0.181; n = 4). However, no change in holding current was observed for GluK2 KO (n = 9) upon bath applying 300 nM KA indicating that the GluK2 is the predominant KAR subunit responsible for mediating the observed KAR current in these neurons. (A) Single experiment. (B) Group data.

KARs have different expression patterns at different synapses and also the composition of subunits vary. In order to determine the role of GluK1 and GluK2 subunit in the KAR mediated current in the LIII mEC neurons, GluK1 and GluK2 KO mice were used. By bath applying 300 nM KA, the holding current in the GluK1 KO changed by 118.13±19.73 pA which was not significantly altered (*p* = 0.181) when compared to the changes observed in wild-type mice (86.82±7.4 pA, n = 4 each for GluK1 KO and WT). However, in the GluK2 KO there was no change in holding current over the whole duration of bath application of 300 nM KA (figure 3A – single cell data; 3B – group data, n = 9 for GluK2 KO). This suggests that GluK2 is the major subunit mediating the KAR currents in the LIII mEC pyramidal neurons.

### Characterization of the KARs

**Figure 4:**
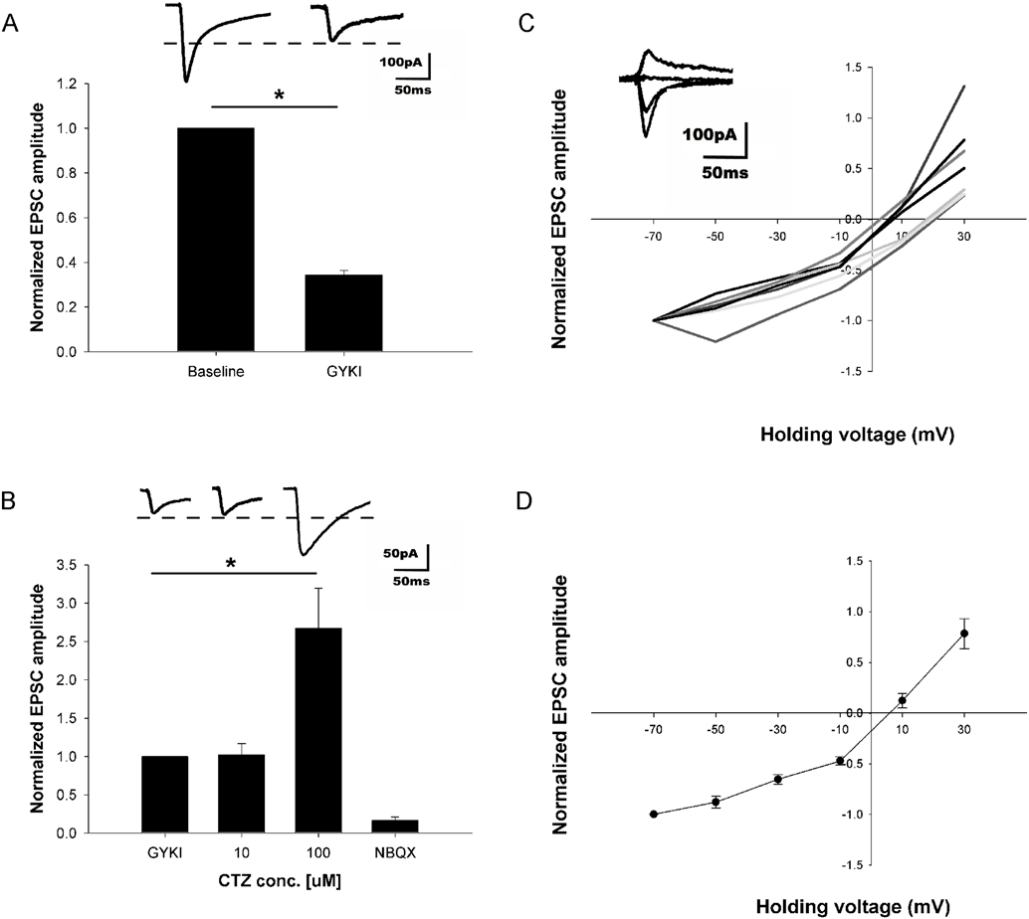
IV curve and characterization of kainate receptors on LIII mEC pyramidal neurons using photolytic uncaging of glutamate. (A) Photolytic uncaging of glutamate at the cell soma elicited inward currents, which were reduced to 34.32% (±2.05%; *p*<0.01; n = 5) of the baseline value in the presence of GYKI (20 µM). (B) At 20 µM of GYKI no residual AMPA current is seen as there is no potentiation of the resultant EPSC upon application of AMPAR desensitization blocker CTZ (10 µM; *p* = 0.956; n = 4). However, at a higher concentration of CTZ (100 µM), the effect of GYKI is antagonized (*p*<0.01; n = 6). In the presence of GYKI, APV and Gabazine, the holding membrane potential was changed in steps of 20 mV from −60 mV to 40 mV and at each step, 5 responses (5 laser flashes with a inter-stimulus interval of 30 seconds) were recorded by uncaging glutamate over the cell soma. (C) The peak current for each individual cell (n = 7) is plotted against the membrane potential along with the corresponding superimposed current traces (inset). (D) Group data (n = 7). A linear relationship between voltage and current, both at negative and positive potentials suggested the KARs on LIII mEC pyramidal neurons to be mostly of the Ca^2+^ impermeable edited form.

RNA editing (Q/R editing) of KARs influences channel properties. The unedited form of the receptor with glutamine (Q) at the Q/R site renders the channel permeable to Ca^2+^ whereas the edited form of the receptor with the positively charged arginine (R) makes it Ca^2+^ impermeable [21], [22], [23]. To determine whether the KARs present on the LIII mEC pyramidal neurons were of the edited or non-edited form, an IV curve was computed by uncaging 200 µM MNI-Glutamate over the cell soma in the presence of 100 µM APV, 2 µM Gabazine and 20 µM GYKI (figure 4C–D). Initially the cell was held at −60 mV and a baseline of stable responses (20 to 25 sweeps, pulse of 5 ms duration at an inter-stimulus interval of 30 seconds) was obtained in the presence of ACSF containing APV and Gabazine only. After washing in GYKI, the isolated KAR current was 34.32% (±2.05%) of the baseline value (figure 4A, n = 5). This remaining current in GYKI were mediated by KARs because they were blocked completely by NBQX (25 µM; data not shown).

For the IV curve of the KARs, the holding membrane potential was changed in steps of 20 mV from −60 mV to 40 mV and at each step, five responses were recorded. Posthoc analysis was performed by averaging these five responses. Calculated junction potential of 10 mV was subtracted from the holding membrane potential. A linear relationship between voltage and current, both at negative and positive potentials (figure 4C–D, n = 7) suggested the KARs on LIII mEC pyramidal neurons to consist mostly of the Ca^2+^ impermeable edited form. To prove that the IV curve was for purely KAR mediated responses, at the end of each experiment, cells were brought back to a holding membrane potential of −60 mV and either 10 or 100 µM Cyclothiazide (CTZ) was added. There was no potentiation of the response on washing in 10 µM CTZ proving that there was no contribution of AMPARs. However, at a higher CTZ concentration (100 µM), the drug antagonizes GYKI [24] and therefore a large potentiation of AMPARs was seen in this case (figure 4B, n = 4 and 6 for 10 µM and 100 µM CTZ respectively). In separate experiments, 10 µM CTZ was added to mixed AMPAR and KAR responses and this concentration was sufficient to potentiate any existing AMPAR component in the response (data not shown).

### Synaptic activation of KARs

**Figure 5:**
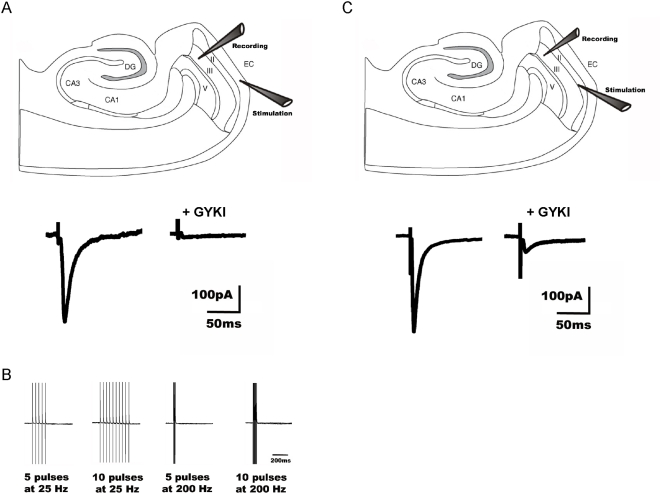
Pathway specific activation of synaptic KARs in LIII mEC pyramidal neurons. (A, B) Recorded LIII mEC pyramidal neurons were held in the whole-cell mode while stimulating the afferent pathway LI mEC. (A, lower panel) In the presence of GYKI (20 µM), no synaptically evoked EPSC_KA_ is detected (n = 8) as seen in an example trace from a single experiment. (B) High frequency stimulations (5 pulses at 25 Hz, 10 pulses at 25 Hz, 5 pulses at 200 Hz and 10 pulses at 200 Hz) were performed in the presence of GYKI. There was no detectable EPSC_KA_ under these stimulation conditions as well indicating the absence of synaptic KARs upon stimulation of this pathway. (C) To determine, whether any other input pathway would yield a significantly higher proportion of synaptic KAR mediated current, we stimulated at the border of LII–III mEC. In the presence of GYKI, an EPSC_KA_ was observed (10.24%±1.1% of baseline; n = 5) as seen in an example trace from a single experiment (C, lower panel). Thus by stimulating a different pathway, a EPSC_KA_ could be evoked on LIII mEC pyramidal neurons, suggesting the existence of synaptic KARs in a pathway specific manner.

To investigate the contribution of synaptic KARs to the observed currents, recorded LIII mEC pyramidal neurons in the whole-cell mode were stimulated by placing a stimulation electrode in LI mEC. In this region, the distal apical dendrites of the LIII mEC pyramidal neurons synapse onto the input pathways from the lateral EC. Baseline EPSC_AMPA+KA_ was recorded in the presence of ACSF containing 50 µM APV, 2 µM Gabazine and 20 µM SCH 50911. After a stable baseline response, 20 µM GYKI was washed in to isolate EPSC_KA_ (figure 5A, n = 8). The EPSC was blocked in GYKI. Since there was no EPSC_KA_ in the recorded neurons, the apparent conclusion was that there were no synaptic KARs activated upon stimulation of this pathway. To verify this finding high frequency stimulations (5 pulses at 200 Hz, 10 pulses at 200 Hz, 5 pulses at 25 Hz and 10 pulses at 25 Hz; [25] were performed in the presence of GYKI. There was no detectable EPSC_KA_ under these stimulation conditions indicating the absence of synaptic KARs in the distal dendritic region (figure 5B).

In a further experiment, synaptic stimulation in LI mEC was combined with glutamate uncaging, thereby recording first a synaptic current and thereafter a somatic current from the same cell while all other conditions remained constant. After washing in GYKI, the somatic current was reduced to 30.96%±4.45% of the baseline value while the synaptic current was blocked (data not shown).

Our data suggests that there is negligible contribution of synaptic KARs upon stimulating LI mEC. However, a KAR current was evoked by uncaging glutamate over the cell soma indicating that the functional KARs could be limited to the somatodendritic region of LIII mEC pyramidal neurons. It has been shown that the distribution of KARs can be pathway specific [25], [26]. To determine, whether any other input pathway would yield a significantly higher proportion of synaptic KAR mediated current, we stimulated at the border of LII–III mEC. When kainate currents were isolated in the presence of GYKI, a GYKI resistant component was seen (10.24%±1.1% of baseline), which was blocked by NBQX (figure 5C, n = 5). Thus by stimulating a different pathway, a EPSC_KA_ could be evoked in LIII mEC pyramidal neurons. In an additional experiment, the stimulation electrode was first placed in LI mEC, thereby evoking no EPSC_KA_. However, relocating the electrode within the same experiment to a second position at the border of LII–III mEC without increasing the stimulation intensity, EPSC_KA_ was evoked in the same cell (14.31%±4.7% of baseline; data not shown).

Taken together, the data suggests the presence of KARs limited to the somatodendritic region of LIII mEC pyramidal neurons (somatic uncaging and LII–III stimulation) and a clear lack of KARs in the distal dendrites.

### Role of GluK2 in network synchrony

Oscillations in the gamma frequency are recordable from the entorhinal cortex in humans and rodents. It was recently shown that the medial entorhinal cortex (mEC) in isolation in vitro generates gamma frequency oscillations in response to kainate receptor agonists [4]. Importantly, these kainate-induced oscillations in vitro had the same horizontal and laminar spatiotemporal distribution as seen in vivo.

**Figure 6:**
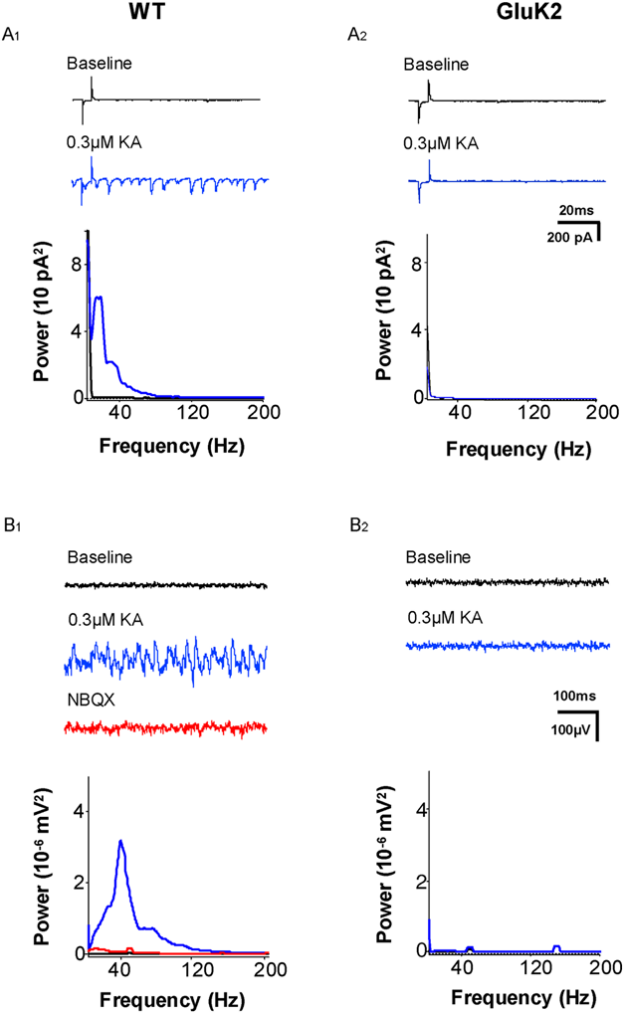
Role of GluK2 in network synchrony. (A_1_) Rhythmic postsynaptic currents were recorded from WT mice (n = 7) following the application of 300 nM KA which had a frequency content of about 10–12 Hz. (A_2_) In comparison such a synchronised increase in spontaneous postsynaptic currents was absent in the GluK2 KO mice (n = 7). (B_1_, B_2_) Local field potential recordings within the superficial layers of the entorhinal cortex were done in an interphase chamber to record KA-induced gamma oscillations. (B_1_) Low concentrations of KA (300 nM) induced robust oscillations (n = 8 slices). Power spectra analysis revealed a major peak frequency of 40 Hz. The KA-induced gamma oscillations were blocked by the KAR/AMPAR-antagonist NBQX. (B_2_) Gamma oscillations were completely abolished in the GluK2 KO mice (n = 7 slices).

We observed during whole-cell patch-clamp recordings from LIII mEC pyramidal neurons, rhythmic postsynaptic currents following the application of low doses of kainate. Figure 6A_1_ shows an example in which under baseline conditions little spontaneous activity was recorded. However, following the application of 300 nM KA a massive increase in spontaneous postsynaptic currents was observed, which had a frequency content of about 10–12 Hz (Figure 6A_1_, n = 7). In comparison such a synchronised increase in spontaneous postsynaptic currents was absent in the GluK2 KO mice upon bath applying KA (fig 6A_2_, n = 7).

Next, we made local field potential recordings within the superficial layers of the entorhinal cortex. These recordings were done in an interface chamber, a condition which improves the reliability and enhances the power of network oscillations. Low concentrations of kainate (300 nM), indeed, induced robust oscillations. Power spectra analysis revealed a major peak frequency of 40 Hz (fig 6B_1_, n = 8 slices). The kainate-induced oscillations in the entorhinal cortex were blocked by the KAR/AMPAR-antagonist NBQX. Further, we again made use of the GluK2 KO mice. Figure 6B_2_ (n = 7 slices) shows that the kainate-induced oscillations were completely abolished in the genetic deletion model.

## Discussion

This study focuses on the layer III of the medial entorhinal cortex, which provides input to the hippocampal CA1 region and subiculum via the perforant path. Since this particular layer suffers from extensive neuronal cell death after epileptic seizures [27], [28], [29], [30], characterization of its synaptic connectivity and modes of synaptic transmission are of critical interest. We show that KAR mediated currents could be evoked in LIII mEC pyramidal neurons. These currents possess properties of Ca^2+^ impermeable KARs and were mediated by GluK2 containing receptors.

KARs can be localized both post- and pre-synaptically. Postsynaptically they facilitate synaptic currents and influence signal integration [31]. Presynaptically they regulate transmission, e.g. by decreasing the probability of transmitter release. Bath application of KA evoked a reversible increase in the holding current, which could also be observed in the presence of the specific AMPAR blocker GYKI 53655, indicating a KAR mediated effect. We determined that at a concentration of 300 nM KA, KARs are selectively activated. Although the subunit composition of individual KARs is not completely clear, ionotropic KARs often include either GluK1 or GluK2 subunits [2], [3], [13], [32]. Using genetically modified mice lacking either GluK1 or GluK2, we have shown unambiguously that GluK2 and not GluK1 subunit contribute to the recorded KAR currents. Since there is considerable presence of transcripts of other kainate receptor subunits (GluK3, GluK4 and GluK5; [12], [33]), one cannot exclude the contribution of heteromeric GluK2 KARs towards the observed kainate current.

Laser mediated glutamate uncaging in the somatic region of LIII mEC pyramidal neurons reliably produced KAR mediated currents in the presence of GYKI and were blocked by the application of NBQX. Additional evidence that these current was mediated exclusively by KAR was provided by the fact that the remaining current was unaltered during application of the AMPAR desensitization blocker CTZ.

The AMPAR subunit GluA2 as well as the KAR subunits GluK1 and GluK2 can undergo Q/R editing, a process determining the calcium permeability, rectification and conductance of the ion channels [21], [22], [23]. Whereas GluA2 editing seems to be almost complete in the adult animal, and disruption of the editing process is lethal [34], [35], KAR editing increases only to a degree of 60–80% during development [21], [22], [23] and interfering with GluK1 editing showed only a mild phenotype [36]. On the other hand, analysis of mutant mice which do not undergo GluK2 editing show NMDAR independent LTP at the EC-DG synapse as well as an increased vulnerability to seizures [37], arguing for a developmental need to down regulate the unedited Ca^2+^ permeable GluK2 receptors and replace them by edited ones.

These considerations, together with the potential role of the EC in epilepsy led us to investigate whether the dominating electrophysiological phenotype of LIII mEC pyramidal neurons is of the unedited, rectifying and Ca^2+^ permeable form or not.

It turned out that the majority of these channels consist of non-rectifying channels, suggesting that the critical receptor subunit is of the edited form. As a functional consequence, a large proportion of these receptors are Ca^2+^ impermeable. Although not very much is known about the physiological role especially of KAR Q/R editing [23], they might play a role in certain pathologic conditions. For example, abnormalities in the ratio of the unedited/edited forms have been reported as a consequence of ischemia [38]. Furthermore, analysis of AMPAR and KAR editing ratio in epileptic patients revealed differences in various brain regions, suggesting a possible involvement of the editing process in this disease [39], [40], [41], [42]. It would be of interest to investigate a potential role of GluK2 editing in the developmental course of epilepsy in the EC.

We tried to evoke synaptic KAR mediated responses by stimulating the inputs from the lateral EC by placing a stimulation electrode in LI mEC. Although we could reliably evoke AMPAR mediated synaptic transmission, which confirmed intact connectivity in our working stimulation conditions, no detectable synaptic current was found after blocking AMPAR mediated currents with GYKI. This argues against synaptically localized KARs, at least in the distal apical dendrite of layer III cells. A similar phenomenon was observed by [25], showing little synaptic transmission after blockade of AMPAR mediated transmission at mossy fibre synapses in the CA3 region of the hippocampus. However, short high frequency stimulation during AMPAR blockade (in the presence of GYKI) in this study lead to a potentiation of the KAR mediated currents. One possible interpretation of these results would be the existence of extrasynaptic KARs that could only be activated by glutamate spillover resulting from synchronous activation of excitatory fibres. To test whether this scenario also holds true for the layer I input to the layer III mEC cells, several similar high frequency protocols were applied. There was no detectable KAR mediated current observed in any case. The most straightforward interpretation of these results is that the distal apical dendrite of layer III cells is devoid of KARs. Also recently, astrocytic glutamate release has been implicated in extrasynaptic activation of neuronal KAR [43], offering a potential explanation and functional role for these receptors, in addition to activation via spillover of synaptically released glutamate after intense stimulation.

However, moving the stimulation electrode to the LII–III border reliably yielded synaptic responses in the presence of GYKI. Based on these results, we conclude that LIII mEC has functional KARs that contain GluK2 and are restricted to the somatodendritic region of the pyramidal neurons in this layer.

Recent studies [44], [45] show that LIII mEC neurons show small but significant residual synaptic currents after blocking AMPAR mediated currents via the application of 100 µM GYKI 52466 by stimulating at the border of LII–III. These responses could be potentiated by brief high frequency stimulation. The present study confirms one of the results obtained there, namely the existence of KAR mediated synaptic currents evoked by LII–III stimulation. Furthermore we extend their findings by identifying the responsible KAR subunit involved, no synaptic KAR current upon LI stimulation and that a KAR current is evoked upon stimulating at the border of LII–III mEC.

We have shown that GluK2 containing KAR-mediated synaptic currents are exclusively restricted to the somatodendritic region of LIII mEC pyramidal neurons, where they are most likely boosting excitatory synaptic transmission, as reported for other synapses [31]. Furthermore, there is connectivity between superficial and deeper layers and within layer III itself [17]. Also LIII mEC neurons project via the perforant path to the CA1 and Subiculum region. Boosting and prolonging the influx of positive charges during stimulation might be an important mechanism influencing the time window required to form long term association between different inputs arriving in this region. The observed localization of KAR mediated currents might also be an explanation for the almost complete loss of LIII mEC pyramidal neurons, often observed in experimental models of epilepsy.

Changes in the power of gamma oscillations have been reported to occur in animal models of psychiatric diseases [5]. Although this is a purely correlative observation, it is interesting to note that these alterations occurred exclusively in the entorhinal cortex and not in the hippocampus. In addition oscillatory behaviour in neuronal circuits might be related to cognitive performance and pathological mental states [46], as suggested by the action of several clinically used drugs. The idea of involvement of KAR in cognitive processes is furthermore supported by genetic studies which have identified mutations in GluK2 as a potential cause for mental retardation [47].

Analysing the cellular and subcellular distribution and properties of these receptors and investigating their functional role in network behaviour such as oscillations will therefore most probably provide insights into underlying physiological mechanisms of cortical and cognitive function and their pathophysiolgogical alterations.

## Supporting Information

Figure S1 Kainate (KA) induced changes in whole-cell holding current of KARs on LII mEC stellate neurons. (A) Electrophysiological and morphological properties of a typical LII mEC stellate neuron. (B) Upon application of 300 nM KA, LII stellate neurons (n = 7) depolarised to a significantly lesser degree as compared to LIII pyramidal neurons (p<0.01; n = 4). (C) Time course data from a single experiment of the whole-cell holding current of a LII stellate neuron upon bath application of 300 nM of KA in the presence of GYKI (20 µM).
(0.63 MB TIF)
