# Correction: GluK2-Mediated Excitability within the Superficial Layers of the Entorhinal Cortex

**DOI:** 10.1371/annotation/40012908-73d7-4bfc-8731-d24ec701dca4

**Published:** 2009-06-05

**Authors:** Prateep S. Beed, Benedikt Salmen, Dietmar Schmitz

The two authors Benedikt Salmen and Dietmar Schmitz should be noted as contributing equally to this work.

