# Supplementary figures and images for: GluK2-Mediated Excitability within the Superficial Layers of the Entorhinal Cortex
Source: PLoS One. 2009 May 18;4(5):e5576. doi: 10.1371/journal.pone.0005576 (PMC2679203; doi:10.1371/journal.pone.0005576)

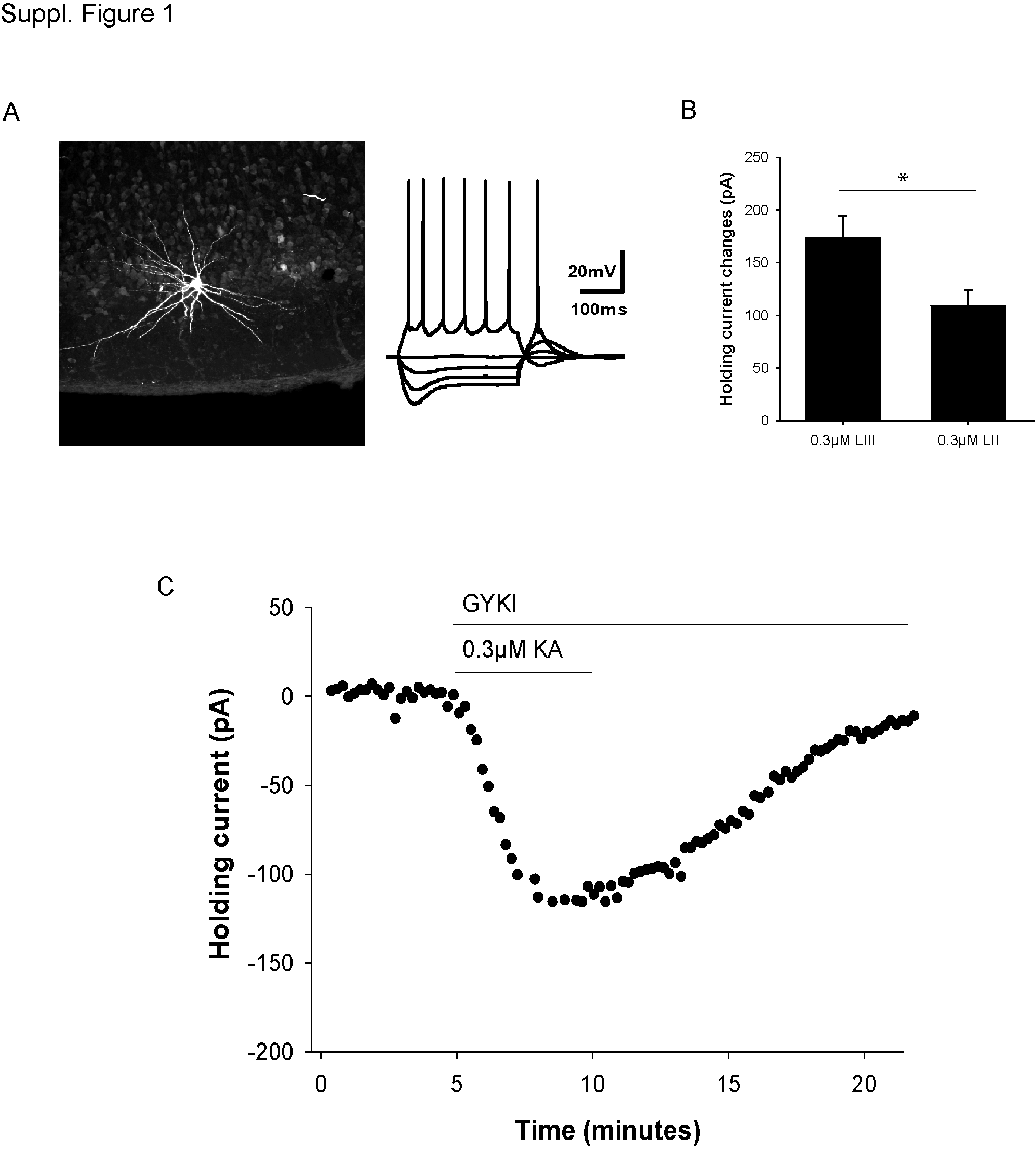

Supplement: Figure S1 — Kainate (KA) induced changes in whole-cell holding current of KARs on LII mEC stellate neurons. (A) Electrophysiological and morphological properties of a typical LII mEC stellate neuron. (B) Upon application of 300 nM KA, LII stellate neurons (n = 7) depolarised to a significantly lesser degree as compared to LIII pyramidal neurons (p<0.01; n = 4). (C) Time course data from a single experiment of the whole-cell holding current of a LII stellate neuron upon bath application of 300 nM of KA in the presence of GYKI (20 µM). (0.63 MB TIF) [file pone.0005576.s001.tif]
